# Load-Carrying Capacity of Double-Shear Bolted Connections with Slotted-In Steel Plates in Squared and Round Timber Based on the Experimental Testing, European Yield Model, and Linear Elastic Fracture Mechanics

**DOI:** 10.3390/ma15082720

**Published:** 2022-04-07

**Authors:** Pavel Dobes, Antonin Lokaj, David Mikolasek

**Affiliations:** 1 Centre for Building Experiments and Diagnostics, Faculty of Civil Engineering, VSB-TU Ostrava, 708 00 Ostrava-Poruba, Czech Republic; 2 Department of Structures, Faculty of Civil Engineering, VSB-TU Ostrava, 708 00 Ostrava-Poruba, Czech Republic; antonin.lokaj@vsb.cz (A.L.); david.mikolasek@vsb.cz (D.M.)

**Keywords:** load-carrying capacity, squared timber, round timber, steel plate, connection, experimental testing, numerical modelling, fracture mechanics

## Abstract

Nowadays, the use of timber as a building material is gaining more prominence. When designing timber structures, it is necessary to pay increased attention to the design of their connections. The commonly used connections are dowel-type connections, which are often used in combination with steel plates slotted into cut-outs in timber members. The presented paper deals with the behavior of double-shear bolted connections of squared timber and round timber with slotted-in steel plates. Several variants of connections with different distances between the fastener and the loaded end were selected for the experimental testing. A total of six types of test specimens were made from spruce timber, for which their selected physical properties were determined and evaluated before the experimental testing. Test specimens of bolted connections were first tested in tension parallel to the grain until failure under quasi-static loading. The connections were broken by splitting. Ductile failure preceded brittle failure. The actual load-carrying capacities were lowest for the lowest end distance. The load-carrying capacities for the middle and the longest end distances were comparable. The results of the experiments were then used for comparison with calculation procedures according to the standard for the design of timber structures and with calculations according to the theory of linear elastic fracture mechanics. The experiments and the analytical models were supported by a simple numerical analysis based on the finite element method.

## 1. Introduction

**Figure 1:**
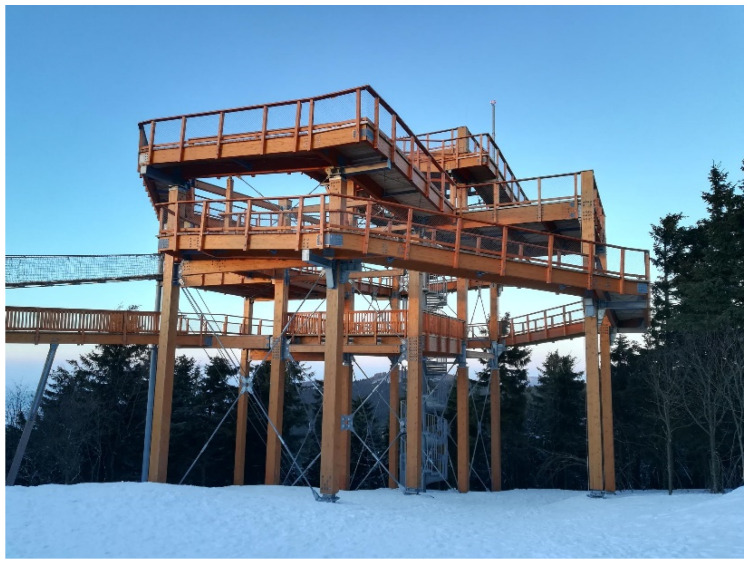
Example of a timber structure with bolted connections.

Nowadays, the use of timber as a building material is becoming more important and popular due to the increasing environmental requirements for construction. Timber is used mainly in the construction of prefabricated houses, multistorey buildings, halls, and more recently, engineering structures, such as bridges, footbridges, and lookout towers (see Figure 1).

When designing timber structures, special attention must be paid to their connections. The load-carrying capacity and stiffness of connections are often decisive for the durability and serviceability of a structure. The influence of the connection stiffness is reflected in the deformation of the structure and the redistribution of internal forces between the connected elements, which fundamentally affects the static design of the whole load-carrying structure. One of the main aims of designers is to design connections where a ductile failure precedes a brittle failure (i.e., to design connections with the highest possible deformation capacity to avoid unexpected failures).

The current European standard for the design of timber structures, Eurocode 5 [1], deals with timber-to-timber or steel-to-timber connections using mechanical fasteners. The most commonly used types of connections are dowel-type connections, which are often used in combination with steel plates slotted into cut-outs in timber elements. Although the standard design of connections is relatively conservative in many respects, it lacks some important knowledge, arising mainly from the requirements of scientific research, but also rapidly evolving construction practice. The standard procedure for determining the load-carrying capacity specifies the ductile failure modes of connections and the corresponding capacities, but does not say anything about the deformation response of the connection to the load at different stages of loading and about actual failure modes. The joint stiffness is approximated only by a simple linear relationship for calculation purposes. Additionally, the distance between the fastener and the loaded end is not taken into account in the calculation procedures. The spacings, edge distances, and end distances are only stated as the minimum required values to avoid the unexpected brittle failure before the ductile failure occurs. In general, very little attention is paid to brittle failure modes and its practical prevention in standards. Probably the biggest drawback is the fact that Eurocode 5 focuses exclusively on squared timber connections and does not offer sufficient support for the design of round timber connections, which are increasingly used due to negligible cutting waste during production.

The presented research deals with the behavior of double-shear bolted connections of squared and round timber with slotted-in steel plates. Several variants of bolted connections with different end distances were selected for experimental testing in order to investigate this influence on the load-carrying capacity and the failure mode. Test specimens of bolted connections were tested by tension parallel to the grain until failure. When describing the behavior of connections in the research, emphasis was placed on the comparison of the experimental testing with calculations according to Eurocode 5 (ductile failure) and also with calculations according to the theory of linear elastic fracture mechanics (hereinafter LEFM) (brittle failure). Numerical modeling was an integral part of the verification of some experimental tests.

## 2. State of the Art

The design of dowel-type connections according to the current Eurocode [1], but also other design codes, is based on the so-called European yield model (hereinafter EYM). EYM assumes ductile failure. Its main ideas were first introduced by the Swedish engineer and scientist Johansen [2]. Initial findings of the EYM were confirmed and further extended by Meyer [3]. Kuenzi [4] presented an idealization that the fastener in contact with the timber acts as a beam on an elastic foundation expressed by the Winkler springs. Although the derived relationships were valid only for the elastic branch of the deformation-load curve of the connections, this work was later used to model dowel-type connections using nonlinear parameters for embedment in timber (e.g., Foschi [5]; Sawata and Yasamura [6]). Doyle [7] observed the positive effect of larger spacing between fasteners on the load-carrying capacity of bolted connections. Mack [8] is the author of a computational model for laterally loaded nail connections, where the deformation response to short-term loads is influenced by many mutually independent factors.

Many scientists are trying to point out the design connections with high ductility, which are not prone to unexpected brittle failure and which, due to their deformation capacity, allow the required redistribution of internal forces within a static system. The ductility of the connection is given by the material properties of timber (density) and fasteners (yield strength and ultimate strength), as well as the geometric parameters of the connection and its individual parts (spacings, end distances, edge distances, location of fasteners, diameter of fasteners, thickness of timber elements), but also other factors (influence of friction, etc.). Whale and Smith [9,10] are the authors of a work that became the basis for a standard relationship for calculating the embedment strength in timber. Ehlbeck and Werner [11] then continued this work. Soltis et al. [12] introduced the term slenderness ratio within dowel-type connections, which is determined by the thickness of the timber element to the fastener diameter (*t*_1_*/d*) and which has a huge influence on a failure mode. The effect of bolt diameter on the load-carrying capacity was also investigated by Daudeville et al. [13]. Smith et al. [14] established an approach for classifying connections based on their ductility (ratio between deformation at yield point and deformation at ultimate point). Muñoz et al. [15] dealt with the correct determination of a yield point from deformation-load curves of connections. Jorissen and Fragiacomo [16] later offered another way to determine ductility in relation to energy dissipation under cyclic loading. Blaß and Shädle [17] compared the ductility of reinforced and nonreinforced dowel-type connections. Sandhaas et al. [18,19] tested double-shear connections with slotted-in steel plates with one, three, or five fasteners parallel to the grain in several different timber species (large density variance). Geiser et al. [20] emphasized the optimization of the postelastic behavior of steel to ensure the required ductility. High ductility of connections is especially desirable for cyclically, dynamically loaded structures [21,22,23,24].

Although the EYM design approach is relatively conservative, it seems to be insufficient to explain the real failure by splitting after reaching ductile failure, or even before reaching this limit for connections with specific geometry and fasteners. The opposite of ductile failure is unwanted brittle failure. Smith and Steck [25] distinguished and described in more detail the various modes of brittle failure. Over time, the approach of classical fracture mechanics began to be used more to describe the brittle failure of connections. Based on a scientific work by Gustafsson [26] and Petersson [27], Jorrisen [28] derived the relationship for the effective number of fasteners *n_ef_* used in standard calculations for multiple fastener connections to include a reduction in load-carrying capacity due to uneven load distribution into the EYM (ductile failure). Quenneville and Mohammad [29] confirmed this hypothesis and showed that the applied load is not distributed evenly across all fasteners, which can lead to a total brittle failure of the connection before the required ductility is reached (especially for stiffer fasteners of larger diameter). Quenneville and Morris [30] presented a simple method for estimating the brittle fracture of dowel-type connections. Smith et al. [31] published a comprehensive publication that deals with fracture and fatigue in timber. Hanhijärvi and Kevarinmäki [32] combined both ductile and brittle failure in their computational procedures. Yurrita and Cabrero focused on the influence of the slenderness of the fastener on the susceptibility to brittle failure in double-shear connections with slotted-in steel plates [33,34,35]. Jockwer and Dietsch [36] also offered an overview of existing design approaches and test results on brittle failure modes. Pavkovic et al. [37] compared experimental tests and numerical models with an analytical calculation according to empirically derived relationships of fracture mechanics (Jorissen [28]; Hanhijärvi and Kevarinmäki [32]). The results confirmed that connections with larger-diameter fasteners are prone to brittle failure. The application of knowledge about fracture mechanics and brittle failure is still under development. The presented research work should also contribute to this trend.

The end distance has an indirect influence on the failure mode of the connection. It is necessary to choose it to prevent an unexpected brittle failure. Many scientific works have been adapted to this. Jorissen [28] tried to describe the distribution of tensile stress perpendicular to the grain and the shear stress in the area of the loaded end as a function of the end distance. César [38] monitored the stress concentration in timber near the hole for the fastener and its influence on the brittle failure. For his experiments on double-shear bolted connections with slotted-in sheet plates, he used end distances that were well below the recommended standard value (2*d*, 3*d*, 4*d*) to focus on brittle failure modes. Dorn et al. [39] varied both the thickness of the timber elements and the end distance (7*d*, 5.5*d*, 4*d*, 2.5*d*). A lower end distance than the required standard value 7*d* led to earlier brittle failure and ductility reduction. Awaludin and Saputro [40] pointed out the possibility of using smaller end distances than the generally accepted standard value. However, this research was carried out for laminated veneer lumber, which, due to production technology, contains fewer imperfections than solid timber. Cui et al. [41] experimentally evaluated the influence of the end distances (5*d*, 6*d*, 7*d*, 8*d*) on the stiffness, load-carrying capacity, and ductility of double-shear dowel-type connections with slotted-in steel plates. The presented research work points out the fact about whether there is any benefit of increasing the end distance above the standard required minimum value with respect to the real load-carrying capacity.

## 3. Theoretical Background

### 3.1. Connections in Timber Structures According to Eurocode 5 Based on EYM

The geometric arrangement of fasteners in a connection (spacings, edge, and end distances) has to meet the minimum values to achieve the expected strength and stiffness. These values should also ensure sufficient ductility and prevention of unexpected brittle failure that could precede EYM ductile failure. For bolts in squared timber connections, a minimum end distance of 7*d* should be respected.

If the load is applied perpendicular to the axis of the fastener (laterally loaded connections), the fastener is embedded into the surrounding timber. The fastener actually acts as a beam loaded by the reaction of the embedded timber. If it is flexible enough, it bends and is able to form one or more plastic hinges. This whole process of interaction must respect the equilibrium of forces and moments.

**Figure 2:**
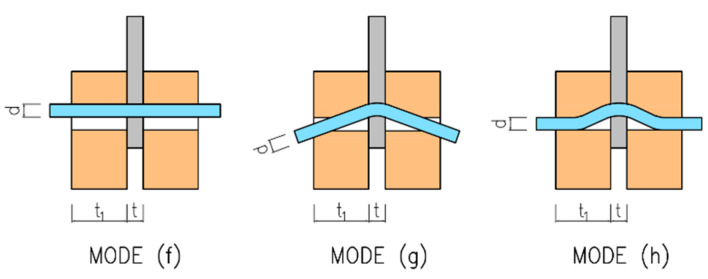
Failure modes for steel-to-timber connections with slotted-in steel plate.

For a double-shear steel-to-timber connection with a steel plate of any thickness as the central member, the characteristic load-carrying capacity of one fastener for one shear plane is determined as the minimum value according to Formula (1) for the failure modes shown in Figure 2.

(1) $$F_{v,Rk}=\left\{\begin{array}{c} f_{h,1,k}\cdot t_{1}\cdot d\;\;\;\;\;\;\;\;\;\;\;\;\;\;\;\;\;\;\;\;\;\;\;\;\;\;\;\;\;\;\;\;\;\;\left(f\right)\\ f_{h,1,k}\cdot t_{1}\cdot d\cdot \left[\sqrt{2+\frac{4\cdot M_{y,Rk}}{f_{h,1,k}\cdot d\cdot t_{1}^{2}}}-1\right]+\frac{F_{ax,Rk}}{4}\;\;\;\;\left(g\right)\\ 2.3\cdot \sqrt{M_{y,Rk}\cdot f_{h,1,k}\cdot d}+\frac{F_{ax,Rk}}{4}\;\;\;\;\;\;\;\;\;\;\;\;\;\;\;\;\;\;\left(h\right) \end{array}\right.$$

where

*F_v,Rk_* is the characteristic load-carrying capacity for a single shear per fastener (N);

*f_h,k_* is the characteristic embedment strength in the timber member (N/mm^2^);

*t_1_* is the smaller of the thicknesses of the timber side member (mm);

*d* is the diameter of the fastener (mm);

*M_y,Rk_* is the characteristic yield moment of the fastener (Nmm);

*F_ax,Rk_* is the characteristic withdrawal capacity of the fastener (N).

**Figure 3:**
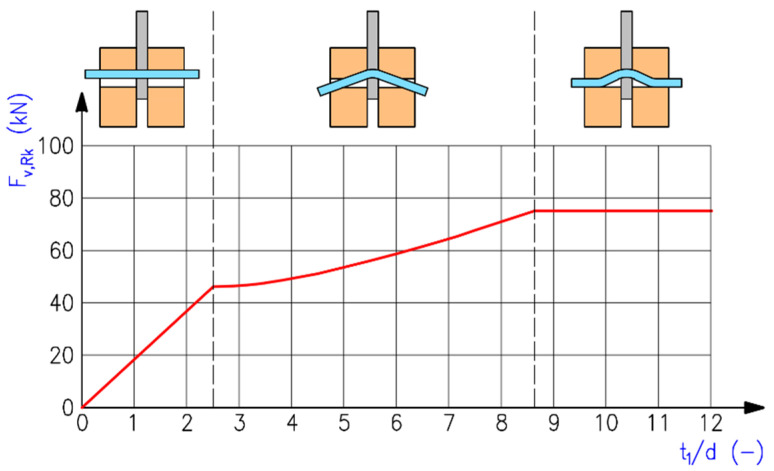
Influence of slenderness ratio *t*_1_*/d* on load-carrying capacity and failure mode of steel-to-timber double-shear connection with slotted-in steel plate (timber C24: *ρ_k_* = 350 kg/m^3^; bolt M20 8.8: *f_u_* = 800 MPa; *d* = 20 mm; *F_ax,Rk_* = 0).

The first term on the right-hand side in the above expressions represents the load-carrying capacity according to Johansen’s EYM (ductile failure modes). The failure mode (f) refers to the embedment in the timber element (typical for fasteners of low slenderness). The failure mode (g) corresponds to the embedment in the timber element together with the formation of one plastic hinge in the fastener. The failure mode (h) corresponds to the embedment in the timber element together with the formation of two plastic hinges in the fastener (typical for flexible fasteners of high slenderness). The slenderness ratio between the thickness of the embedded timber element and the diameter of the fastener *t*_1_*/d* has a huge influence on the failure mode (see Figure 3). The second term on the right-hand side in the above expressions represents the contribution from the rope effect [42], which should be limited to 25% of the Johansen part for bolts. If the contribution from the rope effect is not known, then it should be taken as zero.

For bolts, the characteristic value of the yield moment depends on the bolt diameter d and the characteristic tensile strength *f_u,k_* and is determined according to Formula (2). As can be seen from the units of relevant physical quantities, this formula was derived empirically from the research work of Blaß et al. [43].

(2) $$M_{y,Rk}=0.3\cdot f_{u,k}\cdot d^{2,6}$$

For bolts up to 30 mm diameter, the characteristic embedment strength value in timber parallel to the grain depends on the bolt diameter d and the characteristic density of timber *ρ_k_* and should be calculated according to Formula (3). The basis for this formula was the research work of Whale and Smith [9] and Ehlbeck and Werner [11].

(3) $$f_{h,0,k}=0.082\cdot \left(1-0.01\cdot d\right)\cdot \rho_{k}$$

In the case of multiple-fastener connections, the total load-carrying capacity cannot be determined by a simply sum of full capacities of individual fasteners. These connections are prone to unexpected brittle failure due to block shear. For this reason, it is necessary to reduce the total load-carrying capacity using the reduction factor *n_ef_* ≤ *n*, which was derived from the research work of Jorissen [28].

### 3.2. Connections in Timber Structures According to LEFM

**Figure 4:**
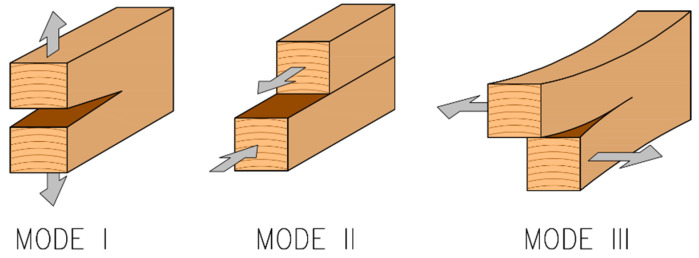
Failure modes according to linear elastic fracture mechanics.

In addition to the standard approach based on EYM, the approach of linear elastic fracture mechanics (LEFM) according to Jorissen [28] can be used. This approach assumes a brittle fracture that occurs as a result of the initiation and gradual propagation of the crack in the contact area of the fastener with the timber. The approach based on LEFM is mainly applied and is often decisive for the failure of dowel-type connections with very stiff fasteners (with a low slenderness ratio *t*_1_*/d*). Fracture mechanics generally recognizes three failure modes (see Figure 4)—mode I (opening mode), mode II (sliding mode), mode III (tearing mode).

The key material property is fracture energy. The initiation of cracks in the contact area is conditioned by exceeding the combination of tensile strength perpendicular to the grain (mode I) and shear strength (mode II). The result is the so-called mixed mode. Mode III does not apply at all in this case. Gustafsson [26] derived relationships for approximate (coefficient of determination *R*^2^ = 0.64) determination of fracture energy for individual modes according to density of timber *ρ*:

(4) $$G_{Ic}=-162+1.07\cdot \rho$$

(5) $$G_{IIc}=3.5\cdot G_{Ic}$$

where

*G_Ic_* is the fracture energy required for mode I (Nm/m^2^);

*G_IIc_* is the fracture energy required for mode II (Nm/m^2^);

*ρ* is the timber density (kg/m^3^).

Petersson [27] described a fracture mechanics model for the mixed mode, which is dependent on the ratio between the tensile stress perpendicular to the grain and the shear stress. According to various sources, the ratio is most often in the range of values *σ_t,_*_90_*/σ_v_* = <0.3; 0.5>. The ratio between the modulus of elasticity perpendicular to the grain and the modulus of elasticity parallel to the grain is considered to be *E*_90_*/E*_0_ ≈ 1/30 for spruce timber of strength grade C24. The fracture energy for the mixed mode *G_c_* is given by the following formulae:

(6) $$G_{c}=\frac{1}{\kappa_{1}}\cdot \left[1+\frac{\kappa_{2}}{2\kappa_{1}}\left(1-\sqrt{1+\frac{4\kappa_{1}}{\kappa_{2}}}\right)\right]$$

(7) $$\kappa_{1}=\frac{1-\kappa_{3}}{G_{IIc}}$$

(8) $$\kappa_{2}=\frac{\kappa_{3}}{G_{Ic}}$$

(9) $$\kappa_{3}=\frac{{\left(\frac{\sigma_{t,90}}{\sigma_{v}}\right)}^{2}}{{\left(\frac{\sigma_{t,90}}{\sigma_{v}}\right)}^{2}+\sqrt{\frac{E_{90}}{E_{0}}}}$$

**Figure 5:**
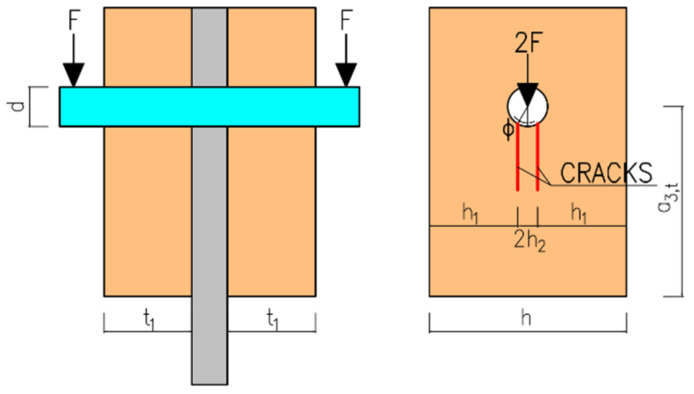
Position of the assumed cracks under the fastener (see also Formula (10)).

It is assumed that two parallel cracks are initiated in both timber parts of the connection in the area under the fastener. The cracks are delimited by a chord of a circular hole with a central angle 2*φ*. The friction angle between the timber and the fastener is considered to be *φ* = 30°. Furthermore, it is necessary to accept approximations that the total load is distributed in both timber parts evenly over a total area of twice 2*h*_2_*·t*_1_ and that the stress is distributed evenly over the thickness of the elements. The assumed position of the cracks is shown in Figure 5.

The load-carrying capacity (fracture force) for one bolt per one shear plane is determined according to Formula (10):

(10) $$F_{LM}=\left(t_{1}+t_{1}\right)\cdot \sqrt{\frac{G_{c}\cdot E_{0}\cdot d\cdot \sin\varphi \cdot \left[h-d\cdot \sin\varphi \right]}{h}}$$

where

*F_LM_* is the load-carrying capacity for a single shear per fastener based on LEFM (N);

*G_c_* is the fracture energy required for the mixed mode (Nmm/mm^2^);

*E_0_* is the modulus of elasticity parallel to the grain (N/mm^2^);

*t_1_* is the thicknesses of one timber side member (mm);

*h* is the width of the timber member (mm);

*d* is the diameter of the fastener (mm);

*φ* is the angle of friction between the timber and the bolt (°).

The modulus of elasticity parallel to the grain of timber *E_0_* (N/mm^2^) can be approximately determined from the density *ρ* (kg/m^3^) on the basis of the empirical Formula (11).

(11) $$E_{0}=48\cdot \rho^{0.91}$$

## 4. Materials and Methods

### 4.1. Experimental Testing

For tension tests parallel to the grain, specimens were made of squared timber with cross-sectional dimensions of twice 60 × 120 mm and round timber with a diameter of 120 mm, which was cut longitudinally. All specimens thus consisted of two identical parts due to the placement of steel plates between the individual parts. The specimens were made of spruce timber of C24 strength grade (determined and verified on the basis of the standards EN 384 [44] and EN 408 + A1 [45]).

**Figure 6:**
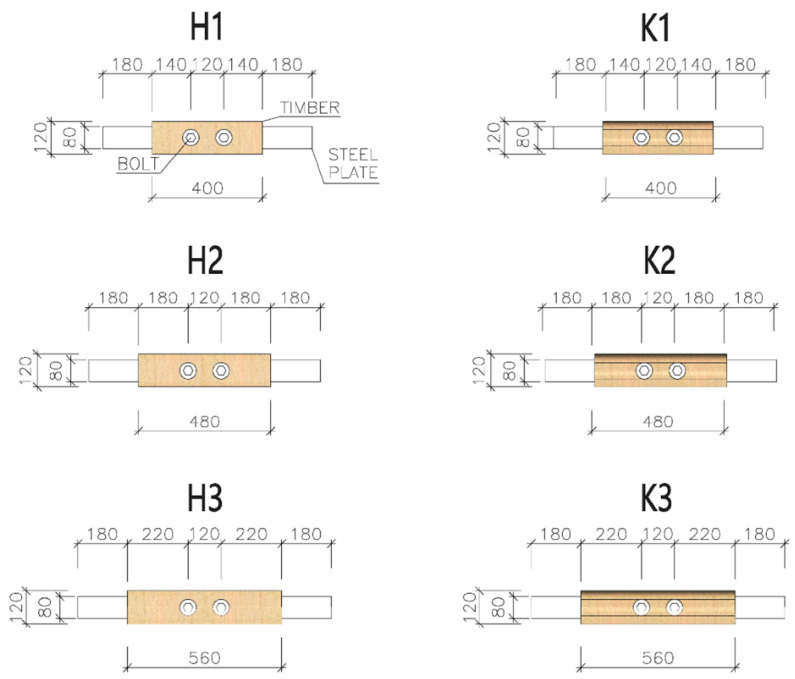
Types of specimens for the experimental testing.

The length of the specimens varied depending on the end distance [46,47]. Specimens of three different lengths were made (see Figure 6)—400 mm (designations K1 and H1), 480 mm (designations K2 and H2), and 560 mm (designations K3 and H3). The circular holes for placing fasteners were created using a drill stand with a drill bit with a diameter of 20 mm.

High-tensile bolts M20, grade 8.8 (yield strength *f_y_* = 640 MPa, ultimate strength *f_u_* = 800 MPa), with a diameter *d* = 20 mm were used as fasteners. The fasteners were placed at three different distances from the loaded end—140 mm (=7*d*), 180 mm (=9*d*), and 220 mm (=11*d*). The axial spacing between two bolts in one specimen was 120 mm.

The slotted-in steel plates with a thickness of 10 mm were made of structural-grade steel S235J0. The dimensions of the steel plates also varied depending on the end distance. It had to be left 180 mm at the protruding end to clamp the steel plates into the jaws of the testing machine. The plates were provided with circular holes with a diameter of 22 mm for the placement of the fasteners (a clearance of 2 mm was left).

In order to properly evaluate the test data, it was necessary to carry out several nondestructive tests immediately before the destructive tests to determine the bulk density and the moisture content of used timber, as these parameters had a significant influence on the mechanical properties of connections.

The moisture content measurement of the timber specimens was performed using a capacitive material moisture meter, Brookhuis FMW-B (Enschede, The Netherlands), with an accuracy of ±0.5%. Scanning to a depth of 20 mm and a correction factor for spruce timber according to its density were set on the device. Four independent measurements were performed on each part of the specimen at different locations, and the resulting moisture content was then given by their arithmetic average (in accordance with the standard EN 13183-3 [48]). The specimens were weighed on a laboratory scale with an accuracy of ±1 g. Their actual dimensions were measured using a tape measure (dimensions over 150 mm) and a digital caliper with an accuracy of ±0.01 mm (dimensions up to 150 mm). Based on the measurements, the bulk density at the actual moisture content was calculated according to the standard ČSN EN 384 [44]; then it was related to a reference moisture content value of 12%.

The experiments were performed using the LabTest 6.1200 electromechanical testing machine from Labortech (Opava, Czech Republic) with 1200 kN maximum electrohydraulic cylinder force. The machine and testing procedure were controlled by computer software.

The specimens were clamped into jaws with special serrations for better adhesion, so the slip in the jaws was eliminated. The lower jaw was static, and the upper jaw was movable. The tested connections were subjected to an axial tensile load with possible additional effects from imperfections for which no significant effect on the evaluated parameters was expected. Possible imperfections include only geometric inaccuracies in the cutting and drilling of the timber specimens. The arrangement and loading of the test specimens were designed to correspond to the actual state of connections in real supporting structures. The aim was not to simulate ideal conditions, but to verify the behavior of the connection under realistic conditions.

**Figure 7:**
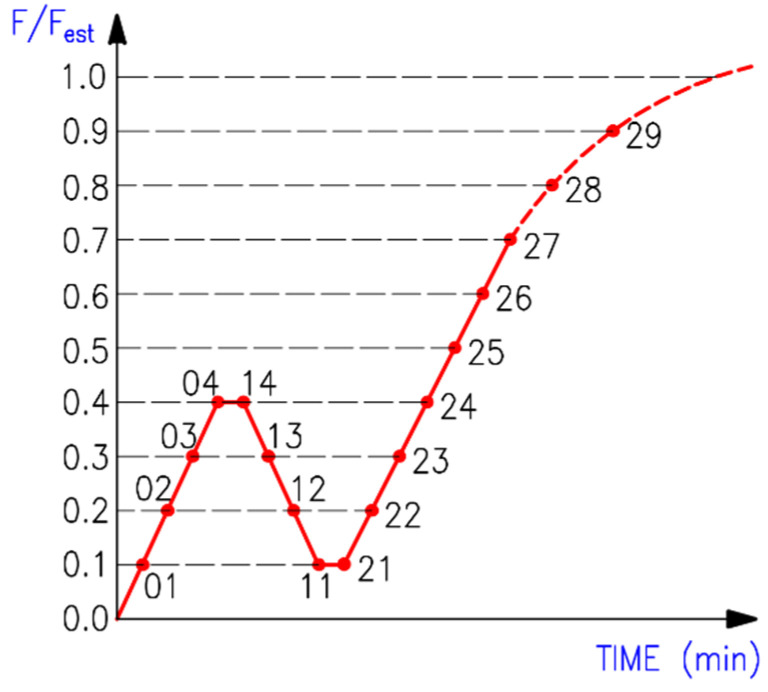
The course of experimental testing.

For this reason, the bolts were also tightened by hand, using a spanner without a specific tightening force, to eliminate the rope effect [42]. The tensile load was generated by electrohydraulic cylinders with a capacity of 1200 kN. During the test, the time, tensile force, and deformations of the connection in the longitudinal direction (i.e., cross-head displacement) were continuously recorded. The loading course (see Figure 7) was carried out in accordance with the standard for testing the connections in timber structures with mechanical fasteners [49]. The following loading procedure was prescribed:

1. Estimation of the maximum force *F_est_* for the tested connection based on experience, calculation, or pretests;
2. Loading of the specimen to 40% of the estimated maximum force, 0.4·*F_est_*, then holding for 30 s;
3. Unloading to 10% of the estimated maximum force, 0.1·*F_est_*, then holding for 30 s;
4. Reloading until the specimen fails.

The loading speed was chosen to be constant (20 kN/min). The total testing time for one specimen was about 10 to 15 min.

### 4.2. Numerical Modelling

Timber is a grown natural anisotropic material. When using numerical methods, it is possible to simply consider orthotropic behavior. It is also considered a homogeneous material and neglects the influence of annual rings, differences between springwood and summerwood, and defects such as knots and cracks.

There are three mutually perpendicular planes of material symmetry, in which timber has specific and independent properties. The planes of symmetry are defined by the longitudinal direction *L* (parallel to the grain), the tangential direction *T* (perpendicular to the grain and tangential to the annual rings), and the radial direction *R* (perpendicular to the grain and perpendicular to the annual rings). The orthotropic material model of timber is then in a system of rectangular coordinates *L*; *T* and *R* with nine different elastic constants, namely, modulus of elasticity in the longitudinal direction *E_L_*; modulus of elasticity in the tangential direction *E_T_*; modulus of elasticity in the radial direction *E_R_*; shear moduli *G* in planes *LT*; *TR* and *LR* and Poisson’s ratios *μ* in planes *LT*; and *TR* and *LR*

The material model of timber within the limits of elasticity was considered to be rectangular orthotropic. The elastic material constants of rectangular orthotropy for spruce timber were taken from the four-point bending test (*E_L_*), were derived according to empirical relationships, or were taken from various sources (Xu et al. [50], *Wood Handbook* [51]), and are given in Table 1. The Hill yield criterion for orthotropic materials was used to predict plastic behavior (Xu et al. [50], Mikolasek [52]).

The elastic-plastic material model with linear strain hardening was used for steel (slotted-in steel plates and bolts together with nuts and washers). The value of the hardening modulus corresponded to 1/10,000 of the modulus of elasticity of the elastic part of the stress–strain curve [52]. The modulus of elasticity in tension and pressure of the isotropic material was taken as *E* = 200 GPa, and Poisson’s ratio *μ* = 0.3. The yield strength for von Mises yield criterion was set to *f_y_* = 235 MPa (plates) and *f_y_* = 640 MPa (bolts).

## 5. Results

**Figure 8:**
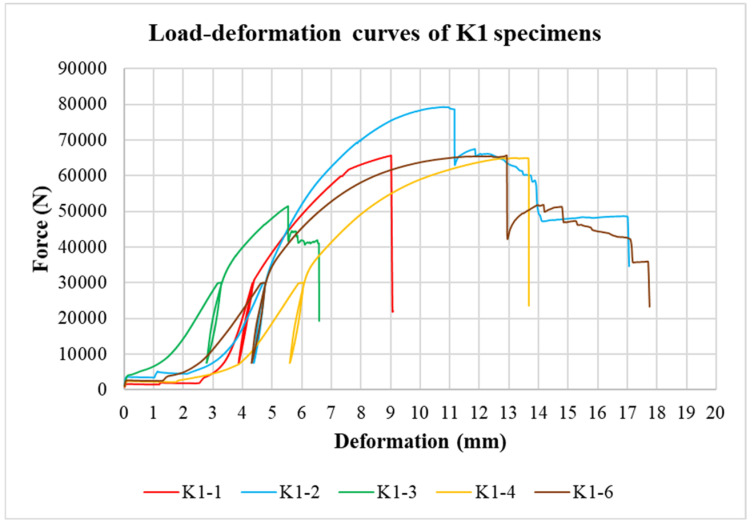
Load-deformation curves of K1 specimens.

**Figure 9:**
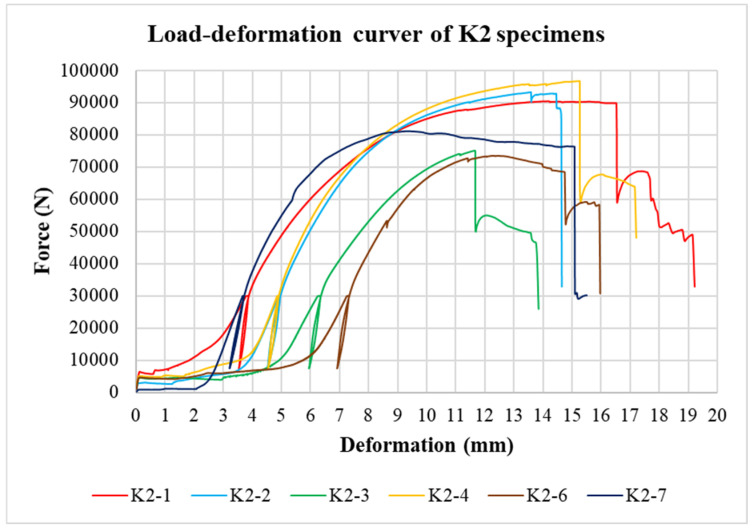
Load-deformation curves of K2 specimens.

**Figure 10:**
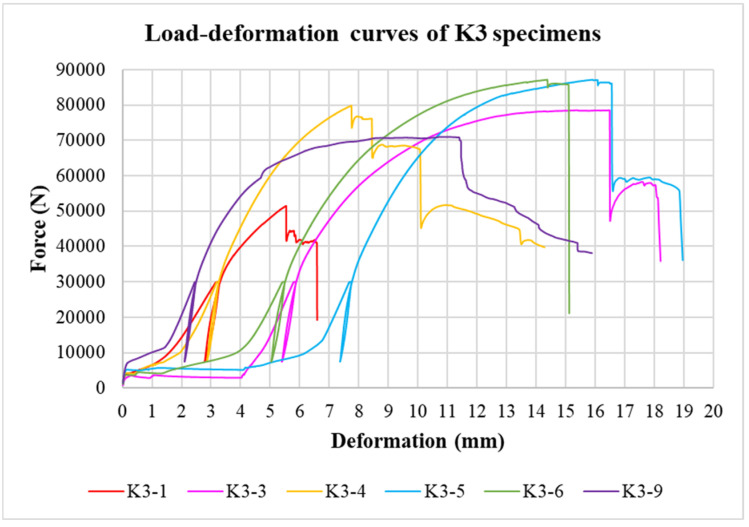
Load-deformation curves of K3 specimens.

**Figure 11:**
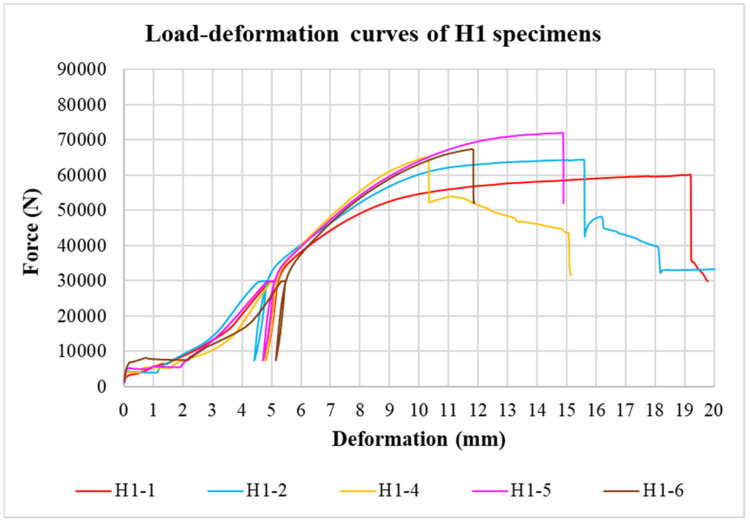
Load-deformation curves of H1 specimens.

**Figure 12:**
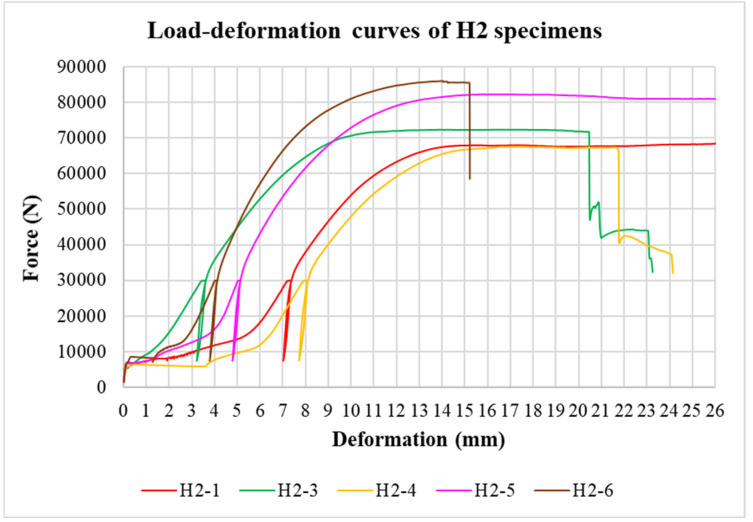
Load-deformation curves of H2 specimens.

**Figure 13:**
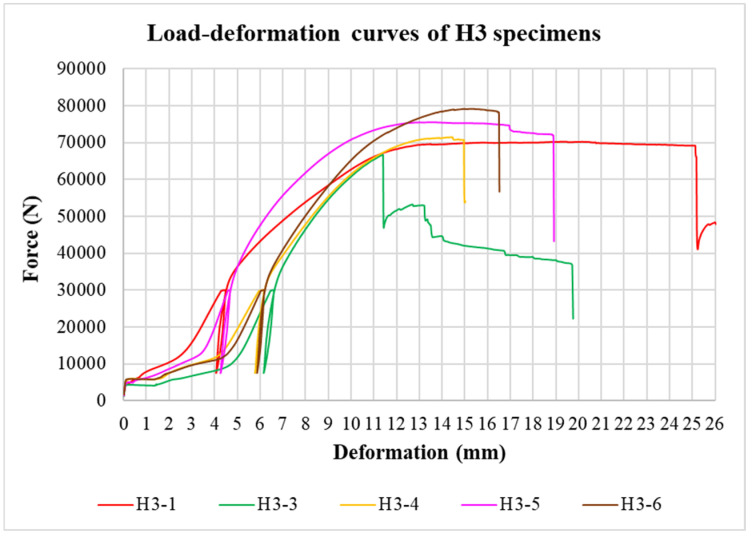
Load-deformation curves of H3 specimens.

The physical properties of timber were determined by nondestructive methods and subsequently evaluated for all tested specimens. Load-deformation diagrams were recorded during the testing to evaluate the load-carrying capacity and the overall behavior of squared and round timber connections with different end distances. The graphs in Figure 8, Figure 9, Figure 10, Figure 11, Figure 12 and Figure 13 show the load-deformation curves of selected specimens of types K1, K2, K3, H1, H2, and H3. The initial load can be seen at 40% of the estimated maximum force, the subsequent unload at 10% of the estimated maximum force, and the reload up to failure. It is also possible to read the maximum load and the corresponding deformation. The graphs are supplemented with tables summarizing the results. Table 2, Table 3, Table 4, Table 5, Table 6 and Table 7 contain selected statistical quantities for moisture content, bulk density, and experimentally determined load-carrying capacity for individual geometric modifications.

**Figure 14:**
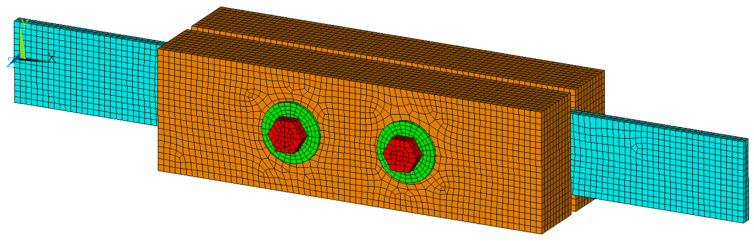
Numerical model and quality of the mesh for H1-type connection in ANSYS Mechanical APDL.

H1-type specimens were selected for the numerical modeling. Numerical analysis was performed using the ANSYS Mechanical APDL software based on the finite element method. The numerical model (see Figure 14) used 3D finite elements, Solid45, with the support of material and geometric nonlinearity. The finite element mesh was divided into several subregions with different finite element sizes with respect to the estimated areas of higher local stress concentrations [53]. A finer mesh was chosen around the contact of the fastener with the timber element. Special contact elements simulating contact pressure and friction in the interface were also modeled. The size of the finite elements did not exceed 6 mm. The total number of finite elements was 36,843.

The nonlinear analysis applied the incremental–iterative Newton-Raphson method. The number of increments of the applied load was 35. The boundary conditions of the numerical model were set in accordance with the clamping and loading of the real specimen in order to achieve the same stiffness model. The possible dynamic effects that could occur during the testing were neglected. The bottom steel plate was firmly fixed in all three directions of displacement at the clamping point, the top steel plate was firmly fixed in two directions of displacement at the clamping point, and the third direction (longitudinal) was left free for deformation-controlled loading.

**Figure 15:**
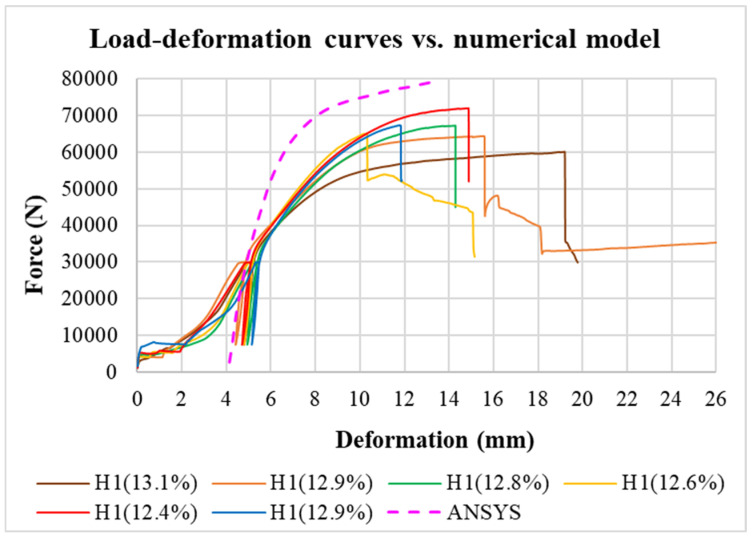
Comparison of real load-deformation curves with the results from the numerical analysis.

The graph in Figure 15 illustrates a comparison of load-deformation curves of real connections and the results from the numerical analysis. The numerical model showed a slightly higher stiffness and load-carrying capacity compared with the experimentally determined curves. It was not possible to take into account the unpredictable initial slip and the gradual increase in stiffness in the initial consolidation phase of the connection when the contact between the timber and the fastener was just forming. The numerical model also did not consider the unloading and reloading cycle.

**Figure 16:**
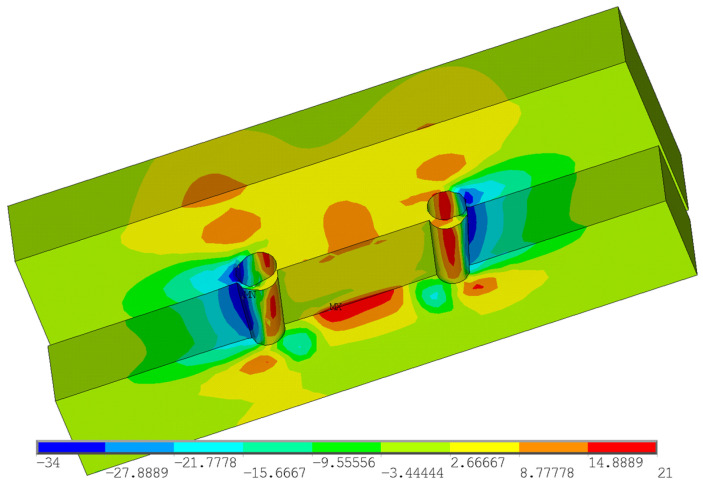
Normal stress in the X direction (tension and compression parallel to the grain).

**Figure 17:**
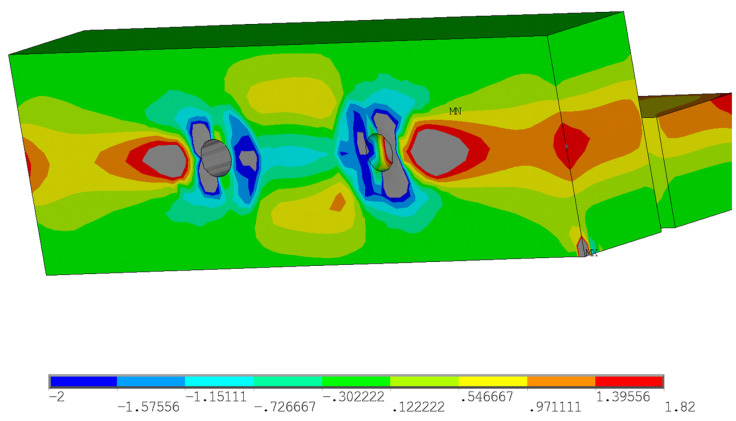
Normal stress in the Y direction (tension and compression perpendicular to the grain).

**Figure 18:**
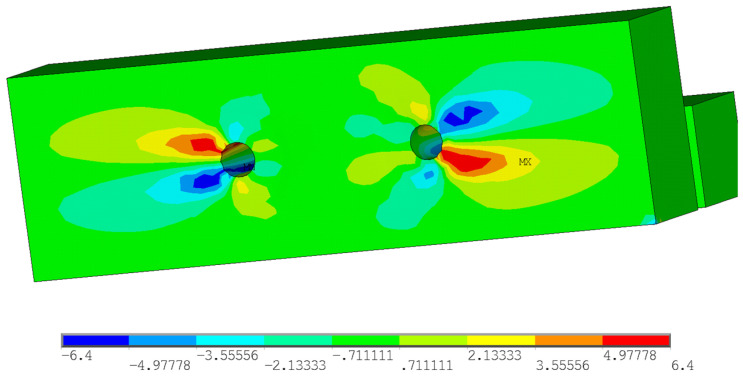
Shear stress in the XY plane (longitudinal shear).

Furthermore, the stress analysis in timber elements was performed. The stress values in MPa are shown on the color scale and correspond to approximately a load of 75 kN. Figure 16 shows the course of normal stress in the X direction. It can be observed that the highest compressive stress parallel to the grain occurs at the loaded end under the bolt (local embedment of the timber around the holes). Tensile stress parallel to the grain occurs between the bolts. Figure 17 shows the course of the normal stress in the Y direction, which represents the stress perpendicular to the grain in the tangential direction. It is obvious that significant peaks occur near the contact of the bolt with the timber. Undesirable tensile stress perpendicular to the grain is visible under the bolt in the area of the loaded end. Figure 18 shows the course of the longitudinal shear stress. It can be observed that the highest shear stress appears under the bolt in two planes, which correspond to the position of the cracks according to the model based on LEFM (see Figure 5).

## 6. Discussion of the Results

**Figure 19:**
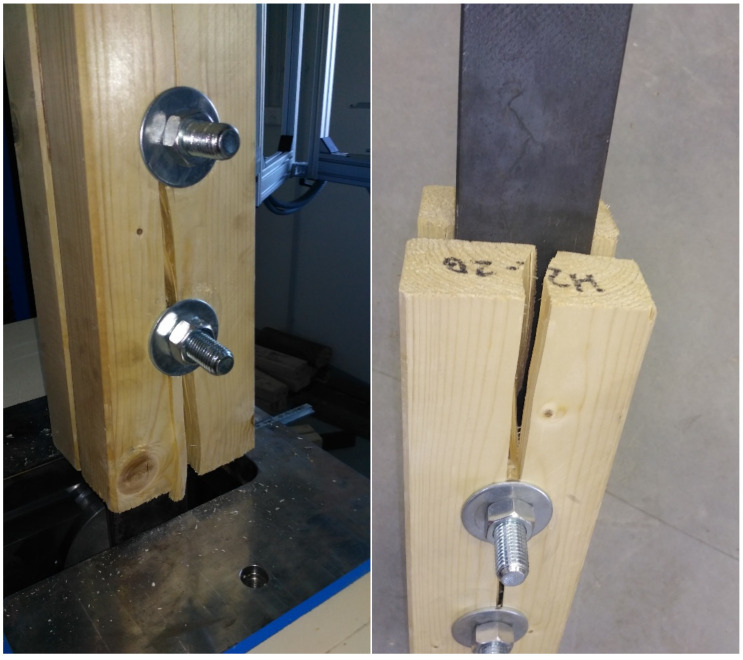
Specimens of squared timber connections after failure.

From the beginning of the loading, the embedment in timber holes increased to the point where the tested specimens were broken by tension perpendicular to the grain. The failure of most specimens occurred by splitting the timber element under the bolt, when the tensile strength perpendicular to the grain was exceeded and when the cross-links between the fibers were broken (see Figure 19). Wood in tension (unlike in compression) shows little plasticity and is broken by brittle fracture. As the failure usually occurred after the visible plastic embedment of the holes, it can be stated that the ductile failure mode precedes the brittle failure mode. The crack propagation was very rapid, usually only in one-half of the specimen under the bolt, in some cases even between the bolts. The other half of the specimen often remained only embedded around the hole. The real failure of the specimens indicated that higher load-carrying capacities could be achieved, which are not limited by Johansen’s ductile failure modes.

In some cases, cracking could be heard in the timber long before the maximum load-carrying capacity was reached, which was caused by the development of microcracks at the microscopic level due to local peak stress, which did not reach a critical size and did not lead to immediate failure. There was only a decrease in current stiffness, followed by stress redistribution, and the connection was still able to carry the increasing load.

**Figure 20:**
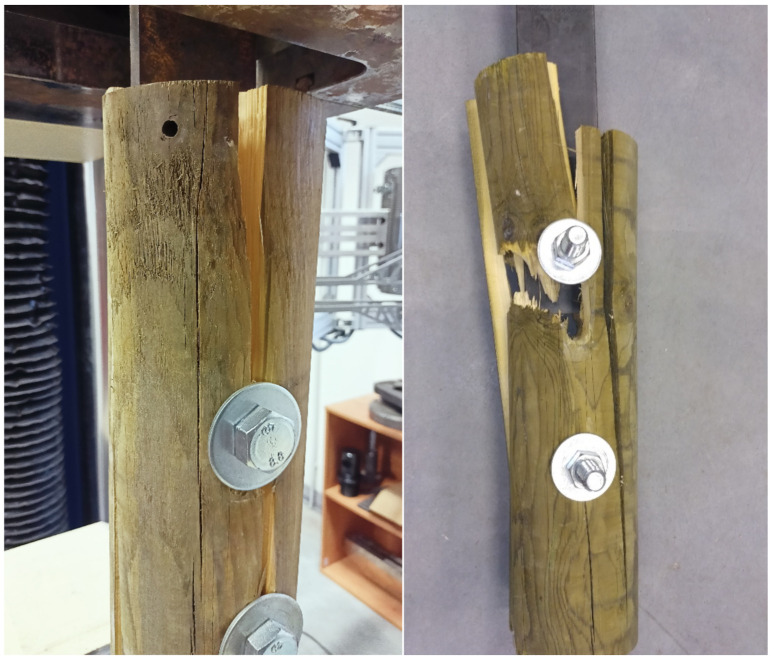
Specimens of round timber connections after failure.

Significant differences in the way of failure could be observed in round timber specimens, where the crack propagation respected the inhomogeneous structure and various natural imperfections of timber (knots, slope of grain, longitudinal shrinkage cracks) and thus looked for the path of least resistance, resulting in cracks with different directions and shapes in individual specimens (see Figure 20). It was not so significant for more homogeneous squared timber. The failure of a few round timber specimens indicated the propagation of two parallel cracks, hence the plug shear. Splitting in combination with a tensile failure parallel to the grain occurred in one round timber specimen, when the element was pushed through the penetrating bolt and part of the cross-section was torn off (see Figure 20). The difference in failure between squared and round timber could also be due to the fact that the thickness of round timber is variable in the width direction, while the thickness of squared timber is constant.

The achieved values of the maximum failure showed that for the lowest end distance (7*d*) they reached the lowest load-carrying capacities. For specimens with higher distances (9*d* and 11*d*), the load-carrying capacities were comparable. It was possible to conclude that with increasing end distance, the actual load-carrying capacity of the connection increases slightly (from the minimum value 7*d* to the value 9*d*). However, as the end distance increases further, the difference is practically negligible (from 9*d* to 11*d*). This was probably due to the fact that at end distances 9*d* and 11*d*, there was no noticeable difference in the distribution of shear stress and tensile stress perpendicular to the grain in the timber element under the fastener (i.e., in the area of the loaded end). Jorrissen [28] theoretically described this stress distribution for the mixed failure mode by splitting in a steel-to-timber double-shear connection with steel plates as the outer members. His research on another type of bolted connection showed that for the end distance greater than 10*d*, the overall stress distribution, concentration, and peaks are practically without difference. Thus, similar findings were also observed for the tested type of connections in this work.

Table 8 shows the average values of the evaluated quantities for individual geometric modifications of the connections. The table contains values of bulk density *ρ_12_*, experimentally determined maximum load-carrying capacity *F_max_*, theoretical load-carrying capacity according to Johansen relationships with substitution of actual bulk density values *F_EC5_* without consideration of the rope effect, load-carrying capacity according to the theory of linear elastic fracture mechanics *F_LEFM_*, ratios between experimental values, and theoretical values. For the comparison of individual types of specimens, the last row shows the maximum load-carrying capacity based on the experiments *F_max,ρ_*, which was normalized according to bulk density. This was a very simplified approximation, where the experimental data were related to the bulk density reference value so that the measured maximum load-carrying capacities Fmax were multiplied by the ratio of the bulk density reference value to the actual bulk density value. The mean density for timber of strength grade C24 *ρ_ref_* = 420 kg/m^3^ was chosen as the reference value.

Experimental data from this research were also supplemented by some results of previous research activities at the Faculty of Civil Engineering, VŠB-TUO [54,55,56,57,58]. The load-carrying capacities of round timber connections were determined experimentally. The tested specimens corresponded to the geometric modification K1. A set of data with similar bulk density values (designation K1^2018^) was selected to compare with this research.

**Figure 21:**
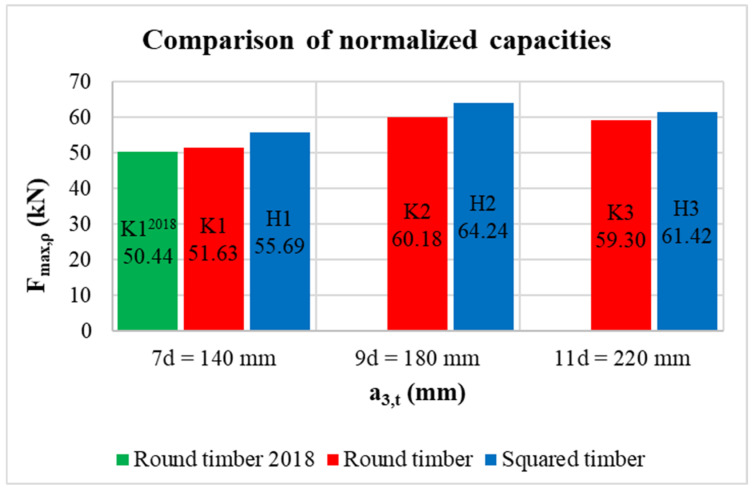
Comparison of the normalized load-carrying capacities for all types of specimens.

The highest values of the actual load-carrying capacities were surprisingly found for specimens with a medium end distance (specimens K2 and H2). The lowest values were found for specimens with the lowest end distance (specimens K1 and H1). The ratios of the capacities for individual geometric modifications were: K2/K1 = 1.16; K3/K1 = 1.11; H2/H1 = 1.14; and H3/H1 = 1.10. Higher values were measured for round timber specimens, which was due to their higher bulk density compared to squared timber specimens (approximately 9% to 13%). After normalization of the data according to bulk density, higher load-carrying capacities were found for squared timber specimens (see Figure 21). In any case, this figure has to be taken as a guide only, because bulk density is not the only factor that governs the load-carrying capacity. The variable thickness of round timber specimens in width directions can also have an effect on this fact. The almost identical (deviation of approximately 2%) load-carrying capacity of specimens K1 and K1^2018^ was also remarkable, which contributed to the mutual verification of two independent research activities at the Faculty of Civil Engineering, VSB-TU Ostrava.

**Figure 22:**
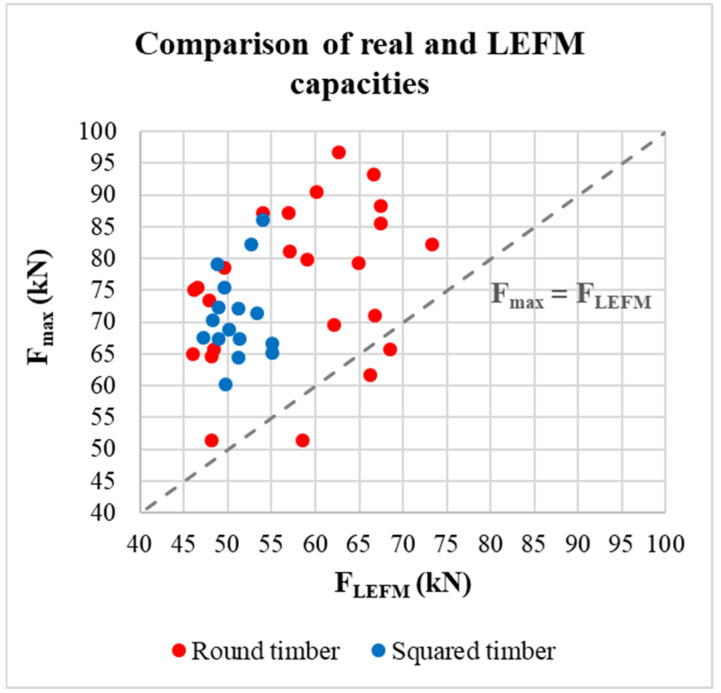
Comparison of the actual load-carrying capacities and the LEFM load-carrying capacities.

All actual load-carrying capacities were significantly higher than standard estimates representing the ductile failure mode according to EYM, which also confirms the hypothesis that the actual total failure of the connections occurred after the ductile failure. A slight effect on the higher load-carrying capacity could also have a possible rope, which could not be completely eliminated due to friction between the individual components of the connection. The evaluation also confirmed that the standard procedures based on EYM can be safely applied to round timber connections. The actual load-carrying capacities were in most cases (apart from three round timber specimens) also higher than the theoretical values according to the theory of LEFM representing brittle failure (see the graph in Figure 22).

**Figure 23:**
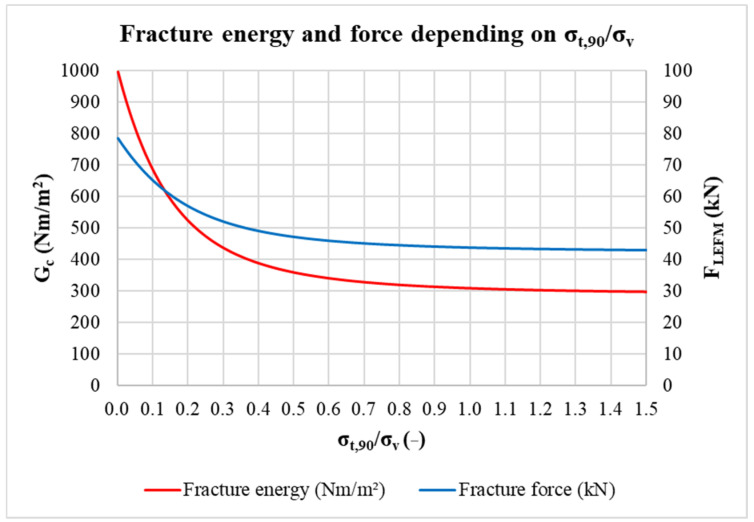
The fracture energy and force depending on the ratio of tensile stress perpendicular to the grain to shear stress.

However, it is necessary to point out that the fracture energy and thus also the fracture force are strongly dependent on the ratio of tensile stress perpendicular to the grain to shear stress. This ratio could not be precisely determined for the calculation, but only estimated. The value from the range of recommended estimates *σ_t,_*_90_*/σ_v_* = 0.3 was taken into the calculation. The influence of this ratio on the value of fracture energy *G_c_* and fracture force *F_LEFM_* for double-shear connection with an in-slotted steel plate (where *t*_1_ = 60 mm; *h* = 120 mm; *d* = 20 mm; *φ* = 30; *ρ* = 420 kg/m^3^; *E*_90_*/E*_0_ = 1/30) is shown in the graph in Figure 23. It is obvious that in the range of values between about 0.1 and 0.6, the differences in the calculated fracture energy and force are relatively significant. Furthermore, it is necessary to keep in mind that the relationships of LEFM for dowel-type connections are only empirically determined approximations (idealized models are subject to error), and the relationships also do not include the length of the loaded end and the resulting stress distribution in it. More accurate data could be obtained using nonlinear models. In fact, the modulus of elasticity of timber does not have a constant value for the entire stress–strain curve until failure, as assumed in the calculations.

**Figure 24:**
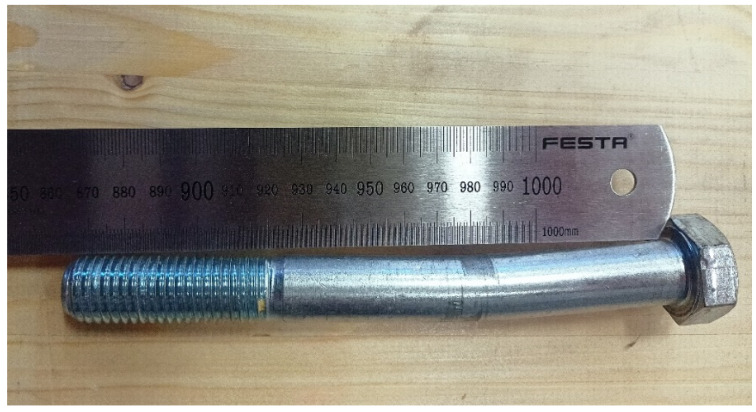
Deformed bolt after the experimental testing.

**Figure 25:**
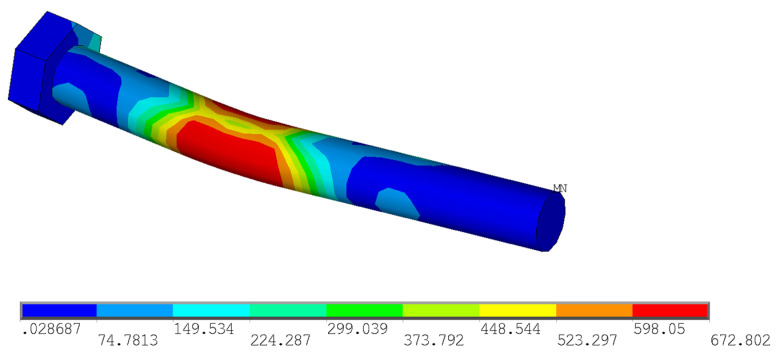
Von Mises stress in the bolt from the numerical analysis.

According to the EYM, the load-carrying capacity of the connection in question is governed by the failure mode (g), which represents the embedment in the timber element together with the formation of one plastic hinge in the fastener (see Figure 2). Deformed bolts were observed rarely in the experiment (only few specimens with higher bulk density) due to the use of relatively brittle high-strength steel (see Figure 24). Figure 25 shows the deformation of the bolt from the numerical analysis for comparison. The stress values in MPa are shown on the color scale and correspond to approximately a load of 75 kN. The maximum von Mises stress *f_max_* = 672.8 MPa is higher than the yield strength *f_y_* = 640 MPa. It would indicate an irreversible plastic deformation of the bolt.

## 7. Conclusions

The presented research dealt with the behavior of double-shear bolted connections in squared timber and round timber with slotted-in steel plates. Several geometric modifications of the connections with different end distances were selected for the experimental testing. The test specimens of the bolted connections were subjected to loading by tension parallel to the grain until failure.

The experimentally obtained data were subsequently evaluated and discussed. The results of quasi-static testing were compared with calculations according to the standard for the design of timber structures, with theoretical models according to the theory of linear elastic fracture mechanics, and with the results of a recent research work at the Faculty of Civil Engineering, VSB-TU Ostrava. Numerical modeling of the selected type of connection was also an important part of the research.

Based on the research, the following conclusions can be drawn:

- The connections were broken by splitting under the bolt, when the tensile strength perpendicular to the grain was exceeded. The brittle failure occurred only after the visible plastic embedment of timber in the area of the hole. Ductile failure thus preceded brittle failure.
- The actual load-carrying capacities were lowest for the lowest end distance (7*d*). For the middle (9*d*) and the longest (11*d*) end distances, the load-carrying capacities were comparable, surprisingly with slightly higher values for the middle end distance. There was no benefit of increasing end distance above 9*d* with respect to the actual load-carrying capacity and ductility of the connection.
- The actual load-carrying capacities were higher than the standard estimates based on Johansen’s EYM. The load-carrying capacities were also higher than the estimates based on the linear elastic fracture mechanics in most cases.

Further research could focus on experimental testing of connections with different geometries and arrangements. On the one hand, it would be appropriate to test connections that would achieve higher ductility before failure (connections with several slender, flexible fasteners with possible reinforcement). These connections would be more suitable for cyclically, dynamically loaded structures. On the contrary, a connection geometry could be used where a brittle failure would precede a ductile failure (connections with very stiff fasteners and small thickness of timber parts). The results of testing such connections could then provide an impetus for deeper research in the field of fracture mechanics.

Of course, it is also desirable to make more comprehensive and accurate numerical models that would provide an important insight into the behavior of different types of connections. Numerical analysis would serve as an effective tool for optimizing the connection geometry before the experiment itself.

## Figures and Tables

**Table 1:** Elastic constants for material orthotropy in ANSYS Mechanical APDL.

Parameter	Value
*E_L_*	9700 MPa
*E_T_*	740 MPa
*E_R_*	470 MPa
*μ_LT_*	0.47
*μ_TR_*	0.25
*μ_LR_*	0.37
*G_LT_*	720 MPa
*G_TR_*	39 MPa
*G_LR_*	750 MPa

**Table 2:** Results of the experimental testing of K1 specimens.

	Moisture Content *w*	Bulk Density *ρ_12_*	Load-Carrying Capacity *F_max_*
**Average value**	12.7%	466 kg/m^3^	68.78 kN
**Minimum value**	11.2%	381 kg/m^3^	51.47 kN
**Maximum value**	13.6%	529 kg/m^3^	85.60 kN
**Standard deviation**	0.9%	64.4 kg/m^3^	12.05 kN
**Coefficient of variation**	7.5%	13.8%	17.5%

**Table 3:** Results of the experimental testing of K2 specimens.

	Moisture Content *w*	Bulk Density *ρ_12_*	Load-Carrying Capacity *F_max_*
**Average value**	12.9%	464 kg/m^3^	79.86 kN
**Minimum value**	11.9%	382 kg/m^3^	61.69 kN
**Maximum value**	14.0%	562 kg/m^3^	96.72 kN
**Standard deviation**	0.8%	63.4 kg/m^3^	12.29 kN
**Coefficient of variation**	5.9%	13.7%	15.4%

**Table 4:** Results of the experimental testing of K3 specimens.

	Moisture Content *w*	Bulk Density *ρ_12_*	Load-Carrying Capacity *F_max_*
**Average value**	12.9%	452 kg/m^3^	76.49 kN
**Minimum value**	12.0%	385 kg/m^3^	51.47 kN
**Maximum value**	14.2%	523 kg/m^3^	88.21 kN
**Standard deviation**	0.7%	51.3 kg/m^3^	11.64 kN
**Coefficient of variation**	5.1%	11.4%	15.2%

**Table 5:** Results of the experimental testing of H1 specimens.

	Moisture Content *w*	Bulk Density *ρ_12_*	Load-Carrying Capacity *F_max_*
**Average value**	12.8%	415 kg/m^3^	66.10 kN
**Minimum value**	12.4%	400 kg/m^3^	60.16 kN
**Maximum value**	13.1%	440 kg/m^3^	72.06 kN
**Standard deviation**	0.2%	13.7 kg/m^3^	3.94 kN
**Coefficient of variation**	1.8%	3.3%	6.0%

**Table 6:** Results of the experimental testing of H2 specimens.

	Moisture Content *w*	Bulk Density *ρ_12_*	Load-Carrying Capacity *F_max_*
**Average value**	12.6%	411 kg/m^3^	75.43 kN
**Minimum value**	11.8%	389 kg/m^3^	67.58 kN
**Maximum value**	13.3%	433 kg/m^3^	86.03 kN
**Standard deviation**	0.6%	18.0 kg/m^3^	8.26 kN
**Coefficient of variation**	5.0%	4.4%	11.0%

**Table 7:** Results of the experimental testing of H3 specimens.

	Moisture Content *w*	Bulk Density *ρ_12_*	Load-Carrying Capacity *F_max_*
**Average value**	13.2%	414 kg/m^3^	72.62 kN
**Minimum value**	12.1%	396 kg/m^3^	66.69 kN
**Maximum value**	14.4%	440 kg/m^3^	79.18 kN
**Standard deviation**	0.9%	19.6 kg/m^3^	4.84 kN
**Coefficient of variation**	6.8%	4.8%	6.7%

**Table 8:** Comparison of the results for all types of specimens.

	K1^2018^	K1	K2	K3	H1	H2	H3
**ρ12 (kg/m^3^)**	451	466	464	452	415	411	414
**Fmax (kN)**	64.99	68.78	79.86	76.49	66.10	75.43	72.62
**FEC5 (kN)**	–	54.80	54.68	53.78	51.24	50.92	51.13
**FLEFM (kN)**	–	58.96	58.70	56.75	51.28	50.59	51.04
**Fmax/FEC5 (–)**	–	1.255	1.460	1.422	1.290	1.481	1.420
**Fmax/FLEFM (–)**	–	1.167	1.361	1.348	1.289	1.491	1.423
**Fmax.ρ (kN)**	60.52	61.96	72.21	71.15	66.83	77.09	73.70

## Data Availability

Data are contained within the article.
